# Toothpick-induced superior mesenteric artery -duodenal fistula with thrombosis: a rare case report

**DOI:** 10.3389/fsurg.2025.1610269

**Published:** 2025-09-02

**Authors:** Zhenlin Sun, Minmin Lu, Xiaorui Ding, Chunxia Xue

**Affiliations:** ^1^ Emergency Department, Binzhou People’s Hospital, Binzhou, Shandong, China; ^2^ Department of Gastroenterology, Binzhou People’s Hospital, Binzhou, Shandong, China

**Keywords:** toothpick, SMA-duodenal fistula, SMA thrombosis, foreign body, case report

## Abstract

**Background:**

Ingestion of foreign bodies, particularly sharp objects like toothpicks, can lead to severe gastrointestinal complications. This case report describes a rare instance of a superior mesenteric artery (SMA)-duodenal fistula with subsequent SMA thrombosis caused by a toothpick.

**Case presentation:**

A 47-year-old woman presented with persistent upper abdominal pain. Abdominal CT revealed increased density around the pancreatic neck. Further CT reconstruction identified a linear foreign body in the duodenum, which was confirmed as a toothpick during surgery. The toothpick had perforated the duodenum and pancreas. Postoperatively, the patient developed lower abdominal pain, melena, and declining hemoglobin levels. Enhanced CT indicated SMA thrombosis. Despite attempts to recanalize the SMA, conservative management was pursued due to good collateral circulation. The patient recovered well and was discharged after stabilization.

**Conclusion:**

This case highlights the potential for toothpick ingestion to cause rare and severe complications, including fistula formation and arterial thrombosis. Clinicians should be vigilant about foreign body ingestion in cases of unexplained abdominal pain and consider comprehensive management strategies to prevent catastrophic outcomes.

## Introduction

Persistent abdominal pain is a common reason for patients to visit gastroenterology and emergency departments, and identifying the underlying cause can be a complex and challenging process. Among the myriad of potential etiologies, ingestion of foreign bodies, particularly sharp objects like toothpicks, is an underappreciated yet significant cause of gastrointestinal complications. These complications may include perforation, abscess formation, and fistula development. While most ingested foreign bodies pass spontaneously without causing harm, a small percentage can lead to severe morbidity (1). Accidental ingestion of toothpicks is more frequently observed in children or intoxicated adults. Once a toothpick enters the digestive tract, it can migrate and lead to various complications, including the formation of different types of fistulas depending on its location (2). This article presents a rare case of a superior mesenteric artery (SMA)-duodenal fistula, followed by the development of SMA thrombosis after surgical removal of the foreign object. We aim to provide insights and experiences for clinicians encountering similar cases.

## Case presentation

A 47-year-old female patient presented to the emergency department with 10 days of persistent dull upper abdominal pain. An abdominal CT scan indicated increased and blurred density in the fat spaces around the neck of the pancreas. Physical examination revealed a soft and flat abdomen, and tenderness in the upper abdomen without rebound tenderness. She was admitted to the gastroenterology department with a preliminary diagnosis of “pancreatitis”. Her medical history included type 2 diabetes mellitus and hypothyroidism.

Upon admission, further tests were conducted: complete blood count showed 76.9% neutrophils, blood glucose was 10.9 mmol/L, while liver and kidney functions, myocardial enzymes, electrolytes, blood lipids, thyroid function tests, blood and urine amylase were all within normal limits. Urological ultrasound, ECG, echocardiography, and chest CT showed no significant abnormalities.

**Figure 1:**
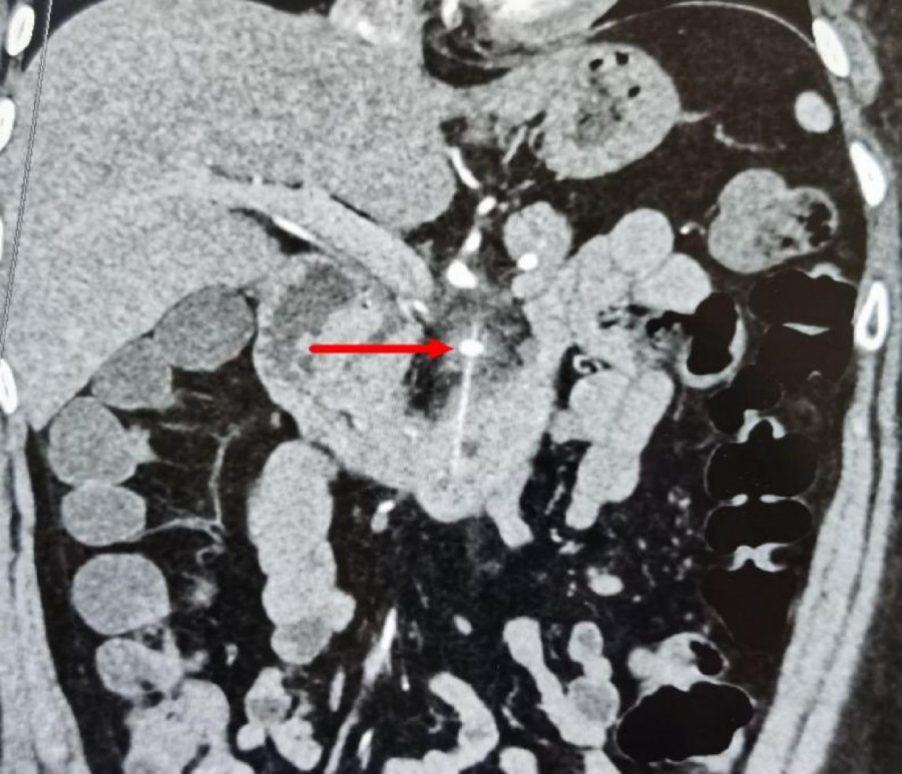
CT reconstruction a linear high-density in the horizontal segment of the duodenum.

Despite acid suppression and fluid therapy, the patient's condition did not improve after three days. A follow-up contrasted abdominal CT, compared with the initial scan, showed a linear high-density mass behind the neck of the pancreas. CT reconstruction (Figure 1) identified a linear high-density foreign body in the horizontal segment of the duodenum, protruding upwards behind the neck and body of the pancreas, with blurred surrounding fatty spaces and exudation. The object was approximately 6.8 cm long, with one end in the duodenal wall area and the other extending to the level of the SMA, closely related to the celiac trunk, with the SMA appearing enlarged and surrounding fat spaces blurred.

**Figure 2:**
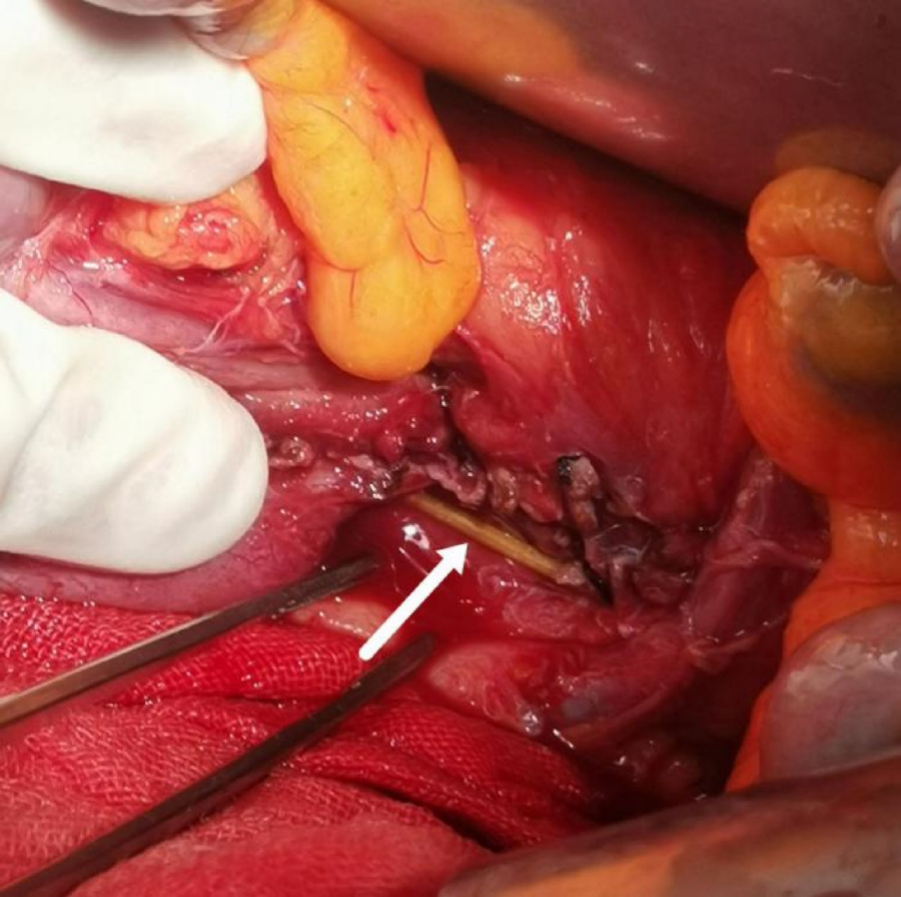
Toothpick was discovered to have perforated the duodenum and the posterior segment of the pancreas.

Further investigation into the patient's history revealed that she had a habit of chewing on toothpicks after meals and had fallen asleep while doing so; this prompted the medical team to perform urgent surgical intervention. During the surgery, the toothpick was discovered to have not only perforated the duodenum but also penetrated the posterior segment of the pancreas, as shown in Figure 2. Significant local edema was observed. The toothpick was carefully removed, and after its removal, the active bleeding from the posterior pancreas was successfully managed using compression hemostasis. Once it was confirmed that there was no further bleeding, the duodenal defect was meticulously repaired without complications, and the procedure was completed successfully.

**Figure 3:**
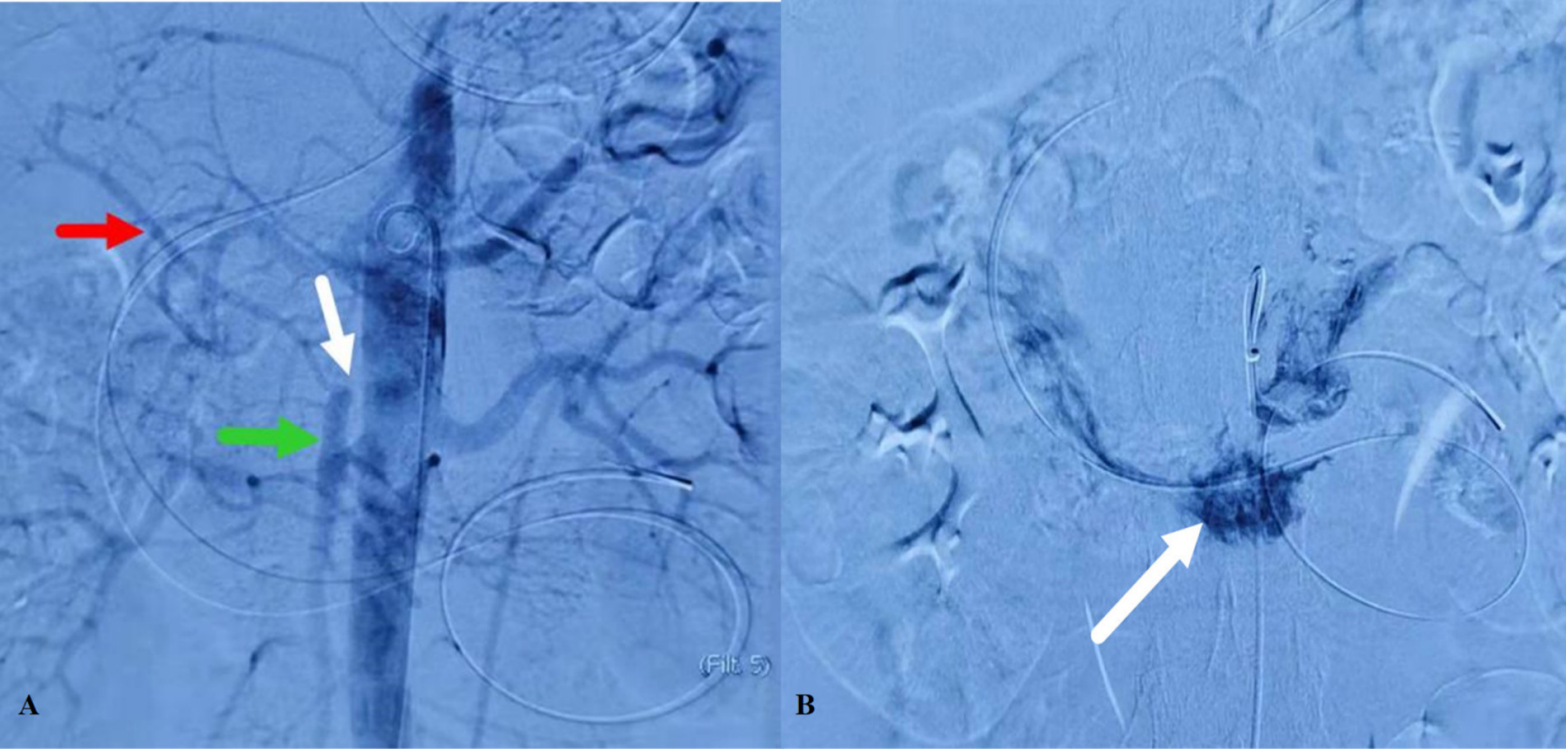
Mesenteric angiography. **(A)** An approximately 2 cm interruption in the proximal trunk of the SMA, with no visualization. **(B)** Contrast agent entering the duodenal lumen.

Postoperatively, the patient's abdominal pain gradually eased; however, beginning on the third postoperative day, she developed new onset intermittent severe lower abdominal colic accompanied by melena and a progressive decline in hemoglobin levels. Enhanced CT of the upper abdomen indicated partial non-visualization of the proximal SMA, suggesting thrombus formation. Emergency interventional treatment was performed, and mesenteric angiography revealed an approximately 2 cm interruption in the proximal trunk of the SMA, with no visualization. The distal SMA was compensated by collateral branches through the gastroduodenal artery, and the distal trunk and its branches appeared normal, with no abnormal staining observed. In the delayed phase, the main trunk of the portal vein was visualized without any filling defects (Figure 3A). The catheter was positioned at the SMA orifice, and a smoke-like appearance indicated contrast agent extravasation, with some of the contrast agent entering the duodenal lumen (Figure 3B).

Attempts were made to recanalize the occluded segment to reach the distal true lumen, but the guidewire could not pass through. Access via the hepatic artery-gastroduodenal artery branch allowed reaching the distal duodenal artery, but retrograde recanalization of the proximal SMA was unsuccessful.

Considering the patient's good collateral circulation compensation, conservative management with antispasmodic, analgesic, circulatory improvement, blood transfusion for anemia correction, and anti-infection therapy was pursued. The patient's abdominal pain gradually subsided, vital signs remained stable, laboratory parameters normalized, and oral intake was well-tolerated. The patient was subsequently discharged in significantly improved condition. The patient underwent regular follow-up for 12 months post-discharge. At the 6-month follow-up, the hemoglobin levels had normalized (12.8 g/dl), and the patient reported no abdominal pain, melena, or coagulation-related complications. By the 1-year follow-up, the patient remained asymptomatic, with stable vital signs and no evidence of recurrent thrombosis or gastrointestinal bleeding. Due to excellent clinical recovery, repeat vascular imaging (e.g., CT angiography) was deemed unnecessary.

## Discussion

Gastrointestinal foreign bodies are a common emergency in gastroenterology, frequently caused by accidental ingestion. Among these, toothpicks are particularly concerning due to their sharp nature and potential to cause serious complications. Toothpicks can penetrate the intestinal wall due to peristalsis and migrate outside the intestines into surrounding tissue spaces or solid organs, most commonly the liver and abdominal cavity (3). There are also reports of toothpicks migrating to major blood vessels, such as arteries and the portal vein (4). These migrations can lead to severe complications, including perforation, bleeding, infection, or even sepsis (5).

The accurate preoperative diagnosis of toothpick ingestion remains a clinical challenge, primarily due to the fact that only a minority of patients can recall the event of swallowing a toothpick. Imaging modalities such as CT, ultrasound, and endoscopy are the primary tools for detecting gastrointestinal foreign bodies. CT is capable of directly visualizing foreign objects; however, the identification of wooden toothpicks is often complicated by their low radiopacity and small size (6). Ultrasound and MRI, while useful in certain contexts, generally exhibit limitations in detecting wooden objects compared to CT. Once a foreign body is diagnosed, it can typically be removed via endoscopy or surgical intervention, depending on the clinical scenario (7).

In the present case, the absence of a documented history of foreign body ingestion from the patient, coupled with the atypical appearance on standard CT cross-sectional images due to the toothpick's unusual intra-body location, initially resulted in a missed diagnosis. The “culprit” was ultimately identified only through CT image reconstruction. In this instance, the toothpick had migrated within the gastrointestinal tract, eventually perforating the duodenum and the SMA, accompanied by a retroperitoneal inflammatory response. The prolonged presence of a toothpick led to local inflammation and structural erosion. Combined with poorly controlled blood sugar levels, this ultimately resulted in the formation of a SMA-duodenal fistula, which was confirmed by angiography. This is the underlying cause of the patient's melena and decreased hemoglobin levels in this case. Aorto-enteric fistulae (AEF) are very rare, occurring between the aorta and its collateral circulation with any part of the bowel (most commonly the duodenum). Arteriography is considered the gold standard for AEF diagnosis. There are only a few global reports of SMA-duodenal fistulas caused by foreign bodies. Shiraev TP (8) reported a case where accidental swallowing of a toothpick led to an iliac artery-duodenal fistula, presenting mainly as gastrointestinal bleeding, with surgical removal of the toothpick.

This case serves as a stark reminder of the critical importance of timely and thorough management of vascular injuries. When a foreign body penetrates a major vessel such as the SMA, the initial focus is often on removing the offending object and achieving hemostasis. However, as demonstrated in this patient, merely removing the foreign body and applying compression may not suffice to prevent catastrophic complications, even in the absence of active bleeding. Notably, mesenteric angiography revealed robust collateral circulation via the gastroduodenal artery, which preserved distal SMA perfusion and intestinal viability without signs of ischemia. This fortuitous vascular compensation not only averted ischemic sequelae but also became the cornerstone of our conservative management strategy for SMA thrombosis. The decision to forgo aggressive intervention was further supported by the patient's hemodynamic stability and the absence of end-organ dysfunction, aligning with contemporary principles of individualized risk-benefit analysis in vascular emergencies (9, 10). Similar cases of SMA thrombosis secondary to foreign body-induced vascular injury are rare but instructive. For instance, Bataev et al. described foreign bodies-induced aortoduodenal fistulas requiring surgical repair, highlighting the variability in management based on etiology and anatomical involvement (11).

The endothelium plays a crucial role in maintaining vascular integrity and preventing thrombosis. Once damaged, as in this case with the toothpick penetrating the SMA, the exposure of subendothelial collagen and release of tissue factors can trigger a cascade of coagulation events (12). The inflammatory response further exacerbates the situation by inducing a hypercoagulable state, creating a “perfect storm” for thrombus formation (13). In such scenarios, it is imperative to recognize that the removal of the foreign body is only the first step. Close attention must be paid to the vascular endothelium and the underlying vessel wall. If there is any suspicion of significant endothelial damage or ongoing inflammation, immediate and aggressive measures should be considered to mitigate the risk of thrombosis. This may include the use of anticoagulant therapy, meticulous vascular repair, or even vascular grafting if the damage is extensive.

In summary, special cases of digestive tract perforation and organ complications caused by foreign body ingestion can provide valuable experience for clinical diagnosis and treatment. Similarly, vascular injuries, particularly those affecting major arteries such as the SMA, are complex and high - risk situations that require a comprehensive and proactive approach. It is crucial to promptly identify endothelial damage and implement appropriate interventions to prevent thrombosis. This case serves as a reminder of the importance of vigilance and thoroughness in managing such high - risk situations.

## Conclusion

The case report presents a rare and complex medical scenario involving the ingestion of a toothpick, leading to a SMA–duodenal fistula and subsequent SMA thrombosis. This case underscores the diagnostic challenges and potential severe complications associated with foreign body ingestion. In cases of unexplained recurrent abdominal pain, clinicians should always consider the possibility of foreign body ingestion.

## Data Availability

The original contributions presented in the study are included in the article/Supplementary Material, further inquiries can be directed to the corresponding author.
